# Preferential Secretion of Thymic Stromal Lymphopoietin (TSLP) by Terminally Differentiated Esophageal Epithelial Cells: Relevance to Eosinophilic Esophagitis (EoE)

**DOI:** 10.1371/journal.pone.0150968

**Published:** 2016-03-18

**Authors:** Prasanna M. Chandramouleeswaran, Dawen Shen, Anna J. Lee, Alain Benitez, Kara Dods, Fiona Gambanga, Benjamin J. Wilkins, Jamie Merves, Yuliana Noah, Sarit Toltzis, Jennifer H. Yearley, Jonathan M. Spergel, Hiroshi Nakagawa, Rene deWaal Malefyt, Amanda B. Muir, Mei-Lun Wang

**Affiliations:** 1 Division of Gastroenterology, Hepatology, and Nutrition, The Children’s Hospital of Philadelphia, Philadelphia, Pennsylvania 19104, United States of America; 2 Department of Pediatrics, Perelman School of Medicine at the University of Pennsylvania, Philadelphia, Pennsylvania 19104, United States of America; 3 Department of Pathology and Laboratory Medicine, Perelman School of Medicine at the University of Pennsylvania, Philadelphia, Pennsylvania 19104, United States of America; 4 Department of Immunology, Merck Research Labs, Palo Alto, California 94304, United States of America; 5 Division of Allergy and Immunology, The Children’s Hospital of Philadelphia, Philadelphia, Pennsylvania 19104, United States of America; 6 Division of Gastroenterology, Department of Medicine, Perelman School of Medicine at the University of Pennsylvania, Philadelphia, Pennsylvania 19104, United States of America; University of Tokyo, JAPAN

## Abstract

Eosinophilic esophagitis (EoE) is a chronic Th2 and food antigen-mediated disease characterized by esophageal eosinophilic infiltration. Thymic stromal lymphopoetin (TSLP), an epithelial derived cytokine which bridges innate and Th2-type adaptive immune responses in other allergic conditions, is overexpressed in esophageal biopsies of EoE subjects. However, the triggers of TSLP expression in the esophageal epithelium are unknown. The objective of the current study was to characterize TSLP expression in human esophageal epithelium in EoE *in vivo* and to determine the role of food antigens upon epithelial TSLP expression *in vitro*. Using immunohistochemistry (IHC), we localized TSLP in esophageal biopsies of active EoE (≥15 eos/hpf), inactive EoE (<15 eos/hpf) and non-EoE control subjects, and found that TSLP expression was restricted to the differentiated suprabasal layer of the epithelium in actively inflamed EoE biopsies. Consistent with these results *in vivo*, inducible TSLP protein secretion was higher in CaCl_2_ differentiated telomerase-immortalized esophageal epithelial cells (EPC2-hTERT) compared to undifferentiated cells of the basal phenotype, following stimulation with the TLR3 ligand poly(I:C). To determine whether food antigens could directly induce epithelial TSLP secretion, differentiated and undifferentiated primary esophageal epithelial cells from EoE and non-EoE subjects were challenged with food antigens clinically relevant to EoE: Chicken egg ovalbumin (OVA), wheat, and milk proteins beta-lactoglobulin (blg) and beta-casein. Food antigens failed to induce TSLP secretion by undifferentiated cells; in contrast, only OVA induced TSLP secretion in differentiated epithelial cells from both EoE and control cell lines, an effect abolished by budesonide and NF-κb inhibition. Together, our study shows that specific food antigens can trigger innate immune mediated esophageal TSLP secretion, suggesting that esophageal epithelial cells at the barrier surface may play a significant role in the pathogenesis of EoE by regulating TSLP expression.

## Introduction

Eosinophilic esophagitis (EoE) is a chronic allergic disease characterized by the infiltration of eosinophils into the esophageal epithelium. Though the etiology of EoE is incompletely understood, both genetic predisposition and environmental triggers are known to play a role in EoE pathogenesis. A unique challenge in EoE is understanding why the inflammatory infiltrate is restricted to the esophageal epithelium, sparing the distal gastrointestinal tract.

Thymic stromal lymphopoietin (TSLP) is an IL-7-like cytokine expressed by multiple cell types, which promotes Th2-type immune responses by influencing dendritic cells (DCs) [1–3]. TSLP is known to drive the inflammatory response in gastrointestinal diseases including celiac disease and inflammatory bowel diseas[4,5] as well as allergic disorders such as asthma[3,6], atopic dermatitis[7] [6], and gastrointestinal allergy[8,9]. Consistent with its role in allergic disorders, TSLP is overexpressed in esophageal biopsies from EoE subjects[10,11] and polymorphisms in TSLP were recently identified in association with EoE[12]. In a murine model of asthma, blockade of TSLP signaling reduced airway inflammation by down-regulating murine DC function[13]. In the context of EoE, it has been proposed that esophageal epithelial-derived TSLP may activate resident dendritic cells which in turn leads to Th2-type polarization. In addition, we recently described a mouse model of EoE-like disease in which esophageal eosinophilic infiltration and subsequent esophageal food impactions were dependent upon TSLP and basophils[11].

Similar to its inducible expression in the airway,[14] TSLP mRNA expression is inducible in human esophageal epithelial cells in response to the Toll-like receptor 3 (TLR3) agonist poly(I:C)[12]. However, to our knowledge there are no published model systems which demonstrate that TSLP protein can be secreted by human esophageal epithelial cells *in vitro*. Establishment of such a model system, including an understanding of the factors that drive esophageal epithelial TSLP protein secretion, would be a powerful tool to study EoE pathobiology *in vitro*.

The precise factors that drive TSLP expression in the esophagus *in vivo* are unknown. Other models suggest that TSLP expression may be specific to disease microenvironments. For example, in atopic dermatitis (AD), epithelial damage promotes TSLP expression in the skin. [15,16]. Similarly, environmental stimuli including diesel exhaust and cigarette smoke induce its expression in bronchial epithelial cells [17].

Food allergies are highly prevalent in EoE, and food antigens are causative in most EoE patients[18], as shown by improvement of EoE patients following restricted [19] or elemental diets [20,21]. Although we and others have identified causative foods in EoE,[19,22] the mechanisms by which food antigens exacerbate EoE remain unknown. Murine models of EoE suggest that, following host immune sensitization, ingested food antigens are absorbed and presented by antigen presenting cells (APCs) to naïve CD4+ T-cells, leading to Th2-type differentiation, eosinophil activation and homing to the esophagus [23],[24] [18,25]. However, animal models have not addressed the mechanism by which ingested food antigens induce esophageal-specific eosinophilic infiltration in EoE.

In allergic conjunctivitis, a disease also affecting a stratified squamous epithelium, TSLP is expressed within the differentiated compartment of the conjunctival epithelium,[26] suggesting that contact with allergens at the barrier surface may play a direct role in regulating TSLP expression. Similar to topical steroids used for allergic conjunctivitis[27], treatment with swallowed steroids with low systemic bioavailability is effective in EoE [28,29], suggesting that the esophageal epithelium may play an inciting role in the EoE inflammatory response.

We hypothesize that differentiated esophageal epithelial cells at the barrier surface play a role in food antigen-mediated inflammation in EoE. In this study, we characterize epithelial TSLP expression patterns in EoE, and demonstrate that terminal differentiation of esophageal epithelial cells is required for maximal inducible secretion of TSLP protein *in vitro*. We demonstrate that esophageal exposure to specific food antigens may contribute to inducible TSLP expression at the barrier surface of the esophagus in EoE, suggesting a novel mechanism by which food antigens initiate EoE-associated inflammation in the esophagus.

## Methods

### Immunohistochemistry (IHC)

Formalin-fixed paraffin-embedded biopsy sections were deparaffinized and boiled in citrate buffer (S1699, Dako, Carpinteria CA). Endogenous peroxidase was quenched with 3% hydrogen peroxide, followed by incubation with anti-TSLP antibody (rat monoclonal GNE01.12F3.B5.4D11, Merck Research Labs, Palo Alto CA), and incubations with biotinylated secondary antibody (712-066-153, Jackson Immunoresearch, West Grove PA), ABC Elite (PK 7100, Vector, Burlingame CA), DAB chromogenic substrate (K3468, Dako) and enhancer (S1961, Dako). Sections were counterstained with Mayer’s Hematoxylin (Poly Scientific, Bay Shore NY), dehydrated, and cover-slipped.

TSLP content was evaluated by a pathologist (BJW) blind to molecular and clinical data on a scale of 0 (no TSLP present) to 3, based on intensity of the stain. Scoring was perfomed on 5 non-EoE, 5 inactive EoE, and 5 active EoE patient biopsies.

### Establishment of primary esophageal epithelial cell lines (EPCs)

All research involving human subjects was approved by the Institutional Review Board at The Children’s Hospital of Philadelphia (CHOP Protocol #7737). Following written informed consent obtained from each subject’s parents or legal guardians, 2–4 additional pinch biopsies were obtained from the distal esophagus during routine diagnostic esophagogastroduodenoscopy (EGD). Primary EPCs were cultivated from these esophageal biopsies using previously published methods. [30] Briefly, biopsies were placed in Hanks BSS buffer, transferred to dispase (0.6ul/ml in PBS), then trypsinized (trypsin-EDTA) for 20 minutes at 37°C. Trypsin was inactivated using soybean trypsin inhibitor (Sigma, St. Louis MO), and biopsies were agitated to release epithelial cells. Cells were pelleted, resuspended, and seeded. Following passage 1, all primary esophageal epithelial cells isolated by these methods exhibited robust expression of the epithelial marker e-cadherin, and lacked expression of mesenchymal markers (not shown), consistent with our previously published study[30].

### Cell culture

Primary EPCs and the nontransformed telomerase-immortalized EPC2-hTERT cell line[31] were cultured in keratinocyte serum-free media (Invitrogen, Grand Island, NY) supplemented with bovine pituitary extract (50ug/ml), epidermal growth factor (1 ng/ml), and penicillin/streptomycin (100units/ml), at 37°C in a humidified 5% CO_2_ incubator. EPC2-hTERT cells were used between passage 30–50, and primary EPCs were used at passage 2–3. Cells were grown in standard KSFM until >90% confluent, then cultured in high calcium-containing KSFM (1.8mM CaCl_2_) for up to 72 hours to promote terminal differentiation.

### Organotypic cell culture (OTC)

OTC models were constructed using previously published methods.[30,32] 5 X 10^5^ EPC2-hTERT cells were seeded onto a collagen matrix containing fetal esophageal fibroblasts. On the fourth day after seeding, epithelial cells were raised to the air-liquid interface and cultured for another 7 days. Cultures were harvested, fixed in 10% neutral buffered formalin, and paraffin embedded for IHC.

### Epithelial cell stimulation

Lyophilized wheat (Greer Labs,Lenoir, NC) was reconstituted in PBS and used at a concentration of 100μg/mL. Chicken egg ovalbumin (OVA) and bovine milk proteins β-lactoglobulin (β-LG) and β-casein were purchased from Sigma and used at concentrations of 1mg/mL (see **S1 Appendix** for dose reponse to OVA). A GenScript (Piscataway, NJ) kit was used for endotoxin quantification. All food antigens had less than 0.003 EU/μg endotoxin. Poly (I:C) (Invivogen, CA) was reconstituted in sterile ddH2O and used at a working concentration of 10 μg/mL.

### RNA isolation and quantitative RT-PCR (qRT-PCR)

RNA was isolated from cell lysates using an RNeasy kit (Qiagen, Valencia, CA) according to manufacturer’s instructions. RNA samples were reverse transcribed using high capacity reverse transcriptase (Applied Biosystems/ABI, Foster City, CA). Taqman expression assays (ABI) for TSLP (assay #Hs01572933_m1, Hs_00263639_m1) was used for qRT-PCR, using the Taqman fast universal PCR master mix kit (ABI). All reactions were performed in triplicate in 96 well plates using a Step One Plus real-time PCR system (ABI). GAPDH (assay #4352934E) was used as an endogenous control to normalize the samples using the ΔΔCT method of relative quantitation, where CT is the threshold cycle.

For evaluation of the short and long isoforms of TSLP (S2 and S3 Appendix), cDNA was isolated using the same methods. We utilized primer sequences described by Fornasa et al[4] and performed quantitative RT-PCR using the Step One Plus real-time PRC system (ABI) as above. Sybr Green was used as the fluorescent probe (ThermoFisher, Grand Island, NY). Β-actin was used as an endogenous control.

### Western Blot

EPC2-hTERT cells were washed with PBS, and lysed with RIPA buffer (1% NP-40, 1% sodium deoxycholate, 0.1% SDS, 0.9% NaCl, 25mM Tris, 1 mM EDTA) containing protease inhibitor cocktail (Sigma). Cells were scraped, and lysates were cleared by centrifugation. Protein concentrations were determined using a BCA protein assay (Pierce Biotechnology, Rockford, IL). Proteins were separated by electrophoresis using NuPage 4–12% Bis-Tris gels (Invitrogen) and transferred to nitrocellulose membranes. Membranes were blocked with 2.5% nonfat dry milk and 2.5% bovine serum albumin overnight at 4°C, incubated with primary antibody, washed in TBST, incubated with secondary antibody, and washed in TBST. Signal was developed using an ECL Western blotting detection kit (Advansta). Mouse anti-involucrin (Sigma, St. Louis MO) was used at a concentration of (1:10,000) and mouse anti-actin (Santa Cruz) was used at a concentration of 1:200. HRP-conjugated anti-mouse (1:5000) (GE Healthcare) was used as a secondary antibody.

### ELISA

TSLP secretion was quantified in cell supernatants using a TSLP ELISA kit (eBiosciences, San Diego, CA) according to manufacturer’s instructions. TSLP concentrations were calculated based upon a standard curve generated by human recombinant TSLP provided by the company. Results were expressed as the mean +/- SEM in pg/mL.

### Human Subjects

The human subjects protocol was approved by the Institutional Review Board at the Children’s Hospital of Philadelphia (CHOP). Esophageal pinch biopsies were obtained during routine diagnostic esophagogastroduodenoscopy (EGD) for isolation of primary EPC lines. Using published guidelines, EoE diagnosis was based upon findings of ≥15 esophageal epithelial eosinophils per high powered field (hpf), basal hyperplasia, absence of non-esophageal eosinophilia, and concurrent high-dose PPI therapy. [18] Subjects on systemic or swallowed corticosteroids were excluded. Subjects with “active” EoE met criteria for EoE, with ≥15 eosinophils per hpf. Subjects with “inactive” EoE were previously diagnosed with EoE, and had improved histology with <15 eosinopils per hpf in the most affected field at the time of sample collection. All control non-EoE subjects had normal biopsies and did not carry a previous diagnosis of EoE, or other chronic inflammatory disease affecting the GI tract. All subjects, including controls, were on proton-pump inhibitor (PPI) therapy for at least 8 weeks prior to endoscopy as prescribed by their primary gastroenterologist.

### Statistical Analysis

Student’s t-test and Kruskal-Wallis test with Dunn’s post comparison were used to analyze data. All tests were two-sided with a significance level of 0.05. All analyses were conducted using Graphpad Prism 5.0 software package.

## Results

### TSLP is overexpressed in the suprabasal compartment of the esophageal epithelium in EoE biopsies with active inflammation, and correlates with markers of terminal differentiation in the esophageal epithelium

**Fig 1:**
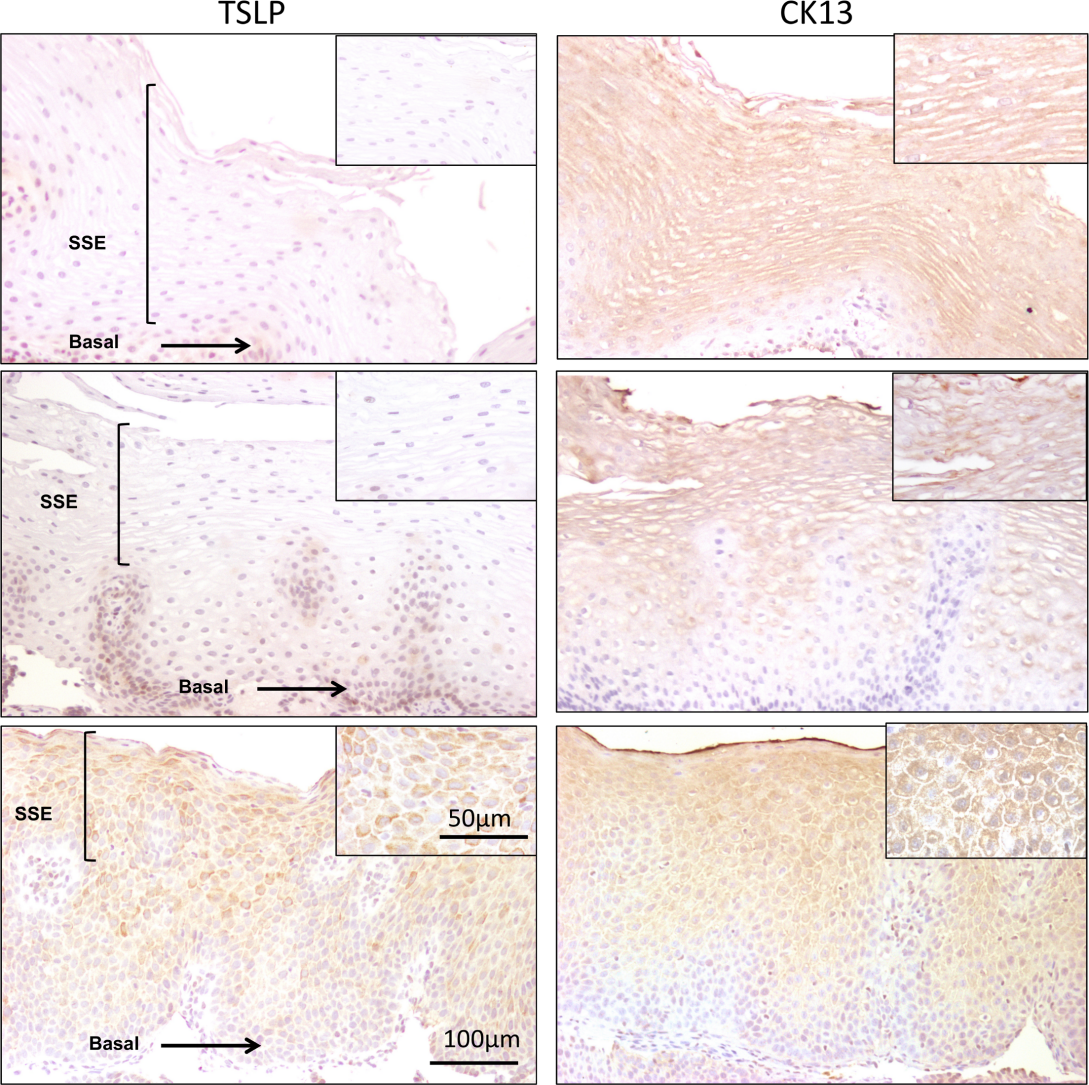
TSLP expression is restricted to the suprabasal differentiated compartment of the esophageal epithelium. Immunohistochemistry for TSLP (A,C,E) and cytokeratin 13 (CK13) (B,D,F) in esophageal pinch biopsies from representative non-EoE control subjects, subjects with inactive EoE (<15 eosinophils per hpf), and active EoE (≥15 eosinophils per hpf). 200X magnification. Larger images represent 100X magnification. The box represents the area chosen for 200X magnification in the smaller side panel. SSE = Stratified squamous epithelium; B = Basal epithelium.

We recently showed that TSLP protein is overexpressed in the esophageal epithelium in actively inflamed human EoE.[11] We used IHC to compare the immunolocalization of TSLP between three groups of pediatric subjects: non-EoE controls, inactive EoE subjects (<15 eos/hpf), and active EoE subjects (≥ 15 eos/hpf). Representative results are shown in **Fig 1.** Non-EoE subjects (**Fig 1A**) and subjects with inactive EoE (**Fig 1C**) expressed little TSLP within the esophageal epithelium (pathologist score average of 1.1 and 0.8 respectively). In contrast, positive TSLP staining was detected in biopsies from patients with active EoE (**Fig 1E**), in which TSLP staining appeared to be limited to the suprabasal, differentiated compartment of the epithelium (pathologist score average of 2.2).

An important histologic feature of EoE is basal cell hyperplasia, secondary to increased cell proliferation in the setting of inflammation. Consistent with this, others have reported that expression of epithelial differentiation markers is reduced in active EoE [33]. To determine whether the observed TSLP staining pattern correlated with markers of epithelial differentiation, we used IHC to immunolocalize cytokeratin 13, a marker of terminal squamous epithelial differentiation [34], in the same tissue sections. CK13 staining was noted throughout the suprabasal compartment of the epithelium in all subjects. Notably, CK13 staining appeared to correlate with TSLP staining in subjects with active EoE (**Fig 1B, 1D and 1F**).

### Calcium induces terminal differentiation of immortalized and primary esophageal epithelial cells *in vitro*

**Fig 2:**
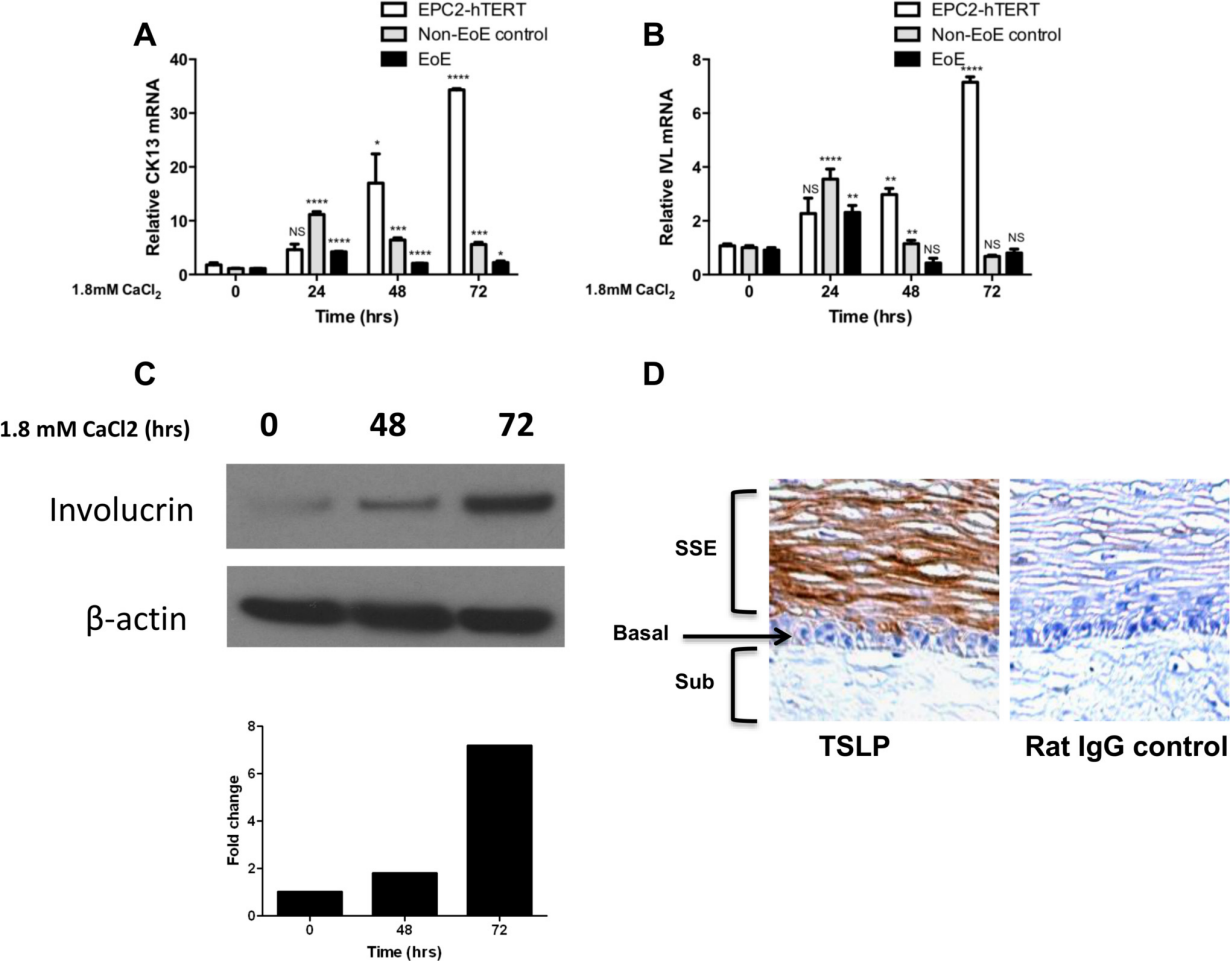
**Calcium induces terminal differentiation of esophageal epithelial cells in-vitro A)** Cytokeratin 13 (CK13) and, **B)** involucrin (IVL), mRNA expression in response to high calcium (1.8mM CaCl_2_) media at 0, 24, 48, and 72 hours. Results are representative of three separate experiments. *p<0.05, **p<0.01, ***p<0.001 and ****p<0.0001 as compared to undifferentiated (0 hr) cells. Immunoblot for **C)** involucrin in calcium differentiated EPC2-hTERT cells. **D**) IHC for TSLP on organotypic cultures of esophageal epithelium. SSE = Stratified squamous epithelium; B = Basal epithelium, Sub = subepithelium.

Our finding that TSLP was localized to differentiated suprabasal epithelial cells in human biopsies prompted us to investigate whether inducible TSLP expression was dependent upon the differentiation state of esophageal epithelial cells *in vitro*. As a first step, we determined the kinetics of squamous epithelial differentiation of human esophageal epithelial cells *in vitro*. It is well established that a calcium gradient exists in stratified squamous epithelium including the epidermis [35], and in the esophageal epithelium [36]. When cultured in standard, low-calcium (0.09 mM) keratinocyte media, telomerase immortalized non-transformed EPC2-hTERT cells maintain phenotypic characteristics of the proliferative basal epithelial phenotype.[31] To induce terminal differentiation, we cultured EPC2-hTERT cells in high calcium-containing media (1.8mM calcium) [37–39] for various time points. Because others have previously suggested that terminal differentiation of esophageal epithelial cells is altered in EoE, we also cultured primary esophageal epithelial cells from EoE and nonEoE subjects under the same conditions. In all 3 cell lines, mRNA expression of terminal differentiation markers cytokeratin 13 (CK13, **Fig 2A**) and involucrin (IVL, **Fig 2B**) was induced in high calcium media. Enhanced esophageal epithelial protein expression of involucrin was confirmed by western blot (**Fig 2C)** in EPC2-hTERT cells.

To explore the correlation between squamous epithelial differentiation and expression of TSLP in physiologic context, we grew EPC2-hTERT cells in organotypic cell culture models, in which esophageal epithelial cells of the basal phenotype are seeded on a matrix of esophageal fibroblasts. Epithelial terminal differentiation and stratification are induced in this model using high concentrations of extracellular calcium and growth at the air-liquid interface, respectively. As shown in **Fig 2D**, TSLP was preferentially expressed in differentiated, suprabasal esophageal epithelial cell in the organotypic context.

### Terminal differentiation of human esophageal epithelial cells enhances poly(I:C)- inducible expression and secretion of TSLP protein *in vitro*

We next determined the effect of terminal differentiation upon inducible epithelial expression of TSLP. Two alternative splice variants of the TSLP gene have been identified. The short-form and long-form TSLP isoforms are distinguished by different methionine initiation codons for protein translation. Harada et al. showed that the TLR3 ligand polyinosinic polycytidylic acid (polyI:C) preferentially induces the expression of the long splice variant of TSLP[40], and Xie et al. previously demonstrated that TSLP protein expression/secretion by human epidermal keratinocytes is dependent on the expression of long-splice form of TSLP[41]. Recently, Fornasa et al described the differential expression of the short and long forms of TSLP, with the short form expressed in steady state conditions, and the long form expressed in inflammatory states within intestine and skin[4]. We therefore compared the expression of total TSLP (which includes both the short and long isoforms) and the long TSLP transcript in differentiated and undifferentiated EPC2-hTERT cells.

**Fig 3:**
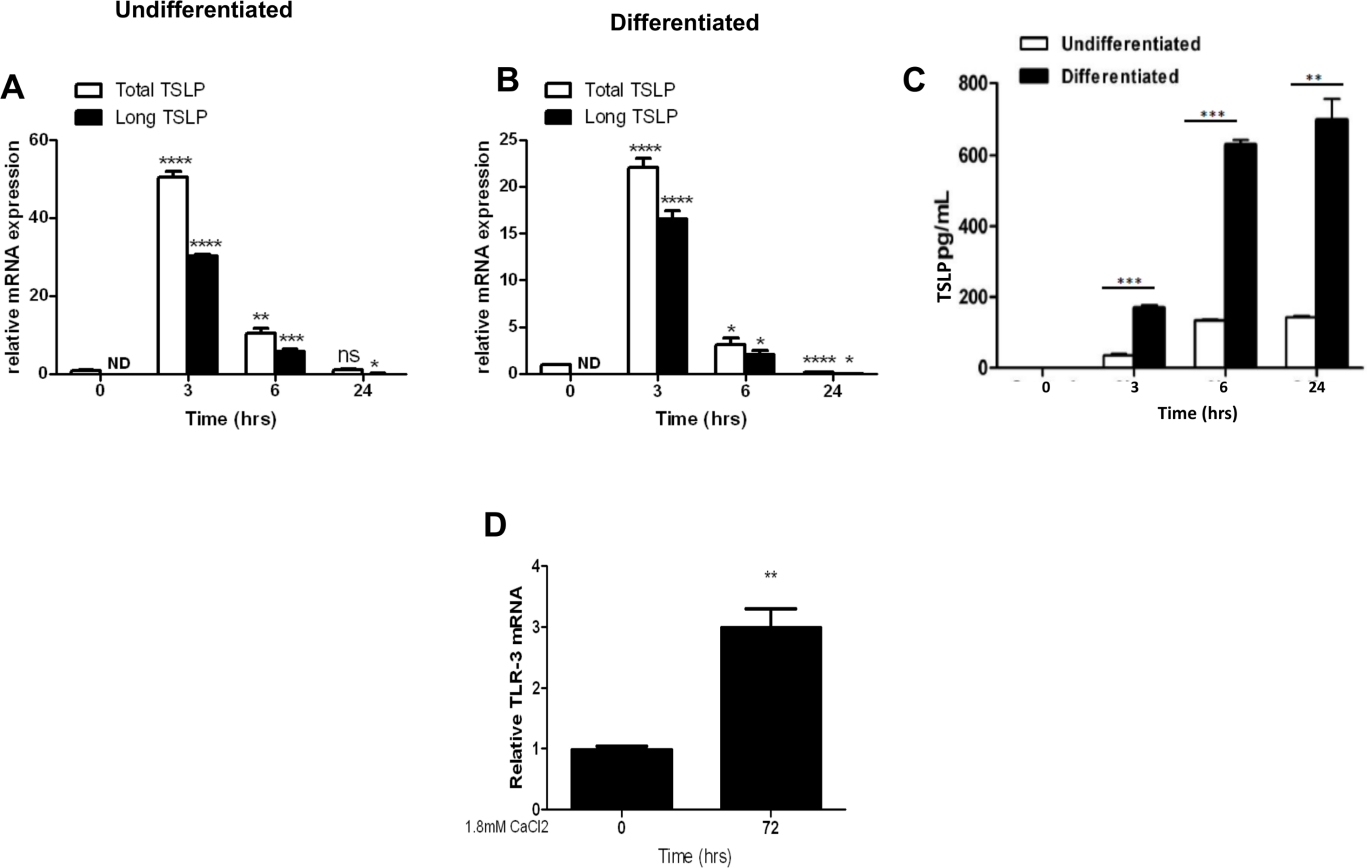
Terminal differentiation of human esophageal epithelial cells enhances the inducible expression and secretion of TSLP protein *in vitro*. TSLP mRNA expression in poly (I:C)-stimulated undifferentiated (**A**) and differentiated (**B**) EPC2-hTERT cells. **C)** TSLP protein secretion (pg/mL) by poly (I:C)-stimulated undifferentiated and differentiated EPC2-hTERT cells. **D**) TLR3 mRNA expression in undifferentiated and differentiated EPC2-hTERT cells. Results are representative of three separate experiments. * p<0.05, p<0.01***p <0.001, p<0.0001 as compared to undifferentiated cells.

In the unstimulated state, TSLP transcript expression was detected in both undifferentiated and differentiated EPC2-hTERT cells, although the long splice isoform was undetectable in both (**Fig 3A and 3B**). Stimulation of undifferentiated and differentiated EPC2-hTERT cells with the TLR3 ligand poly(I:C) induced TSLP transcript expression, with the long TSLP isoform comprising the majority of TSLP transcript at 3hrs and 6hrs post stimulation. Notably, maximal secretion of TSLP protein occurred at 6 and 24 hours following stimulation, which was significantly greater in differentiated EPC2-hTERT cells compared to undifferentiated cells (**Fig 3C**). To determine whether the enhanced effect of poly(I:C) upon differentiated cells was secondary to altered TLR3 expression, we quantified TLR3 mRNA expression in calcium differentiated EPC2-hTERT cells and observed a significant increase in TLR3 mRNA expression following 72 hours of differentiation (**Fig 3D**). Importantly, calcium-mediated terminal differentiation did not alter the constitutive expression or secretion of TSLP in EPC2-hTERT cells (data not shown).

We then compared at the relative expression of the short and long transcripts in the differentiated epithelial cells. We confirmed that the long isoform is the predominant form and its expression peaks at 3–6 hours of stimulation (**S2 Appendix**).

### Specific food antigens induce TSLP secretion by differentiated primary EoE and non-EoE human esophageal epithelial cells

**Fig 4:**
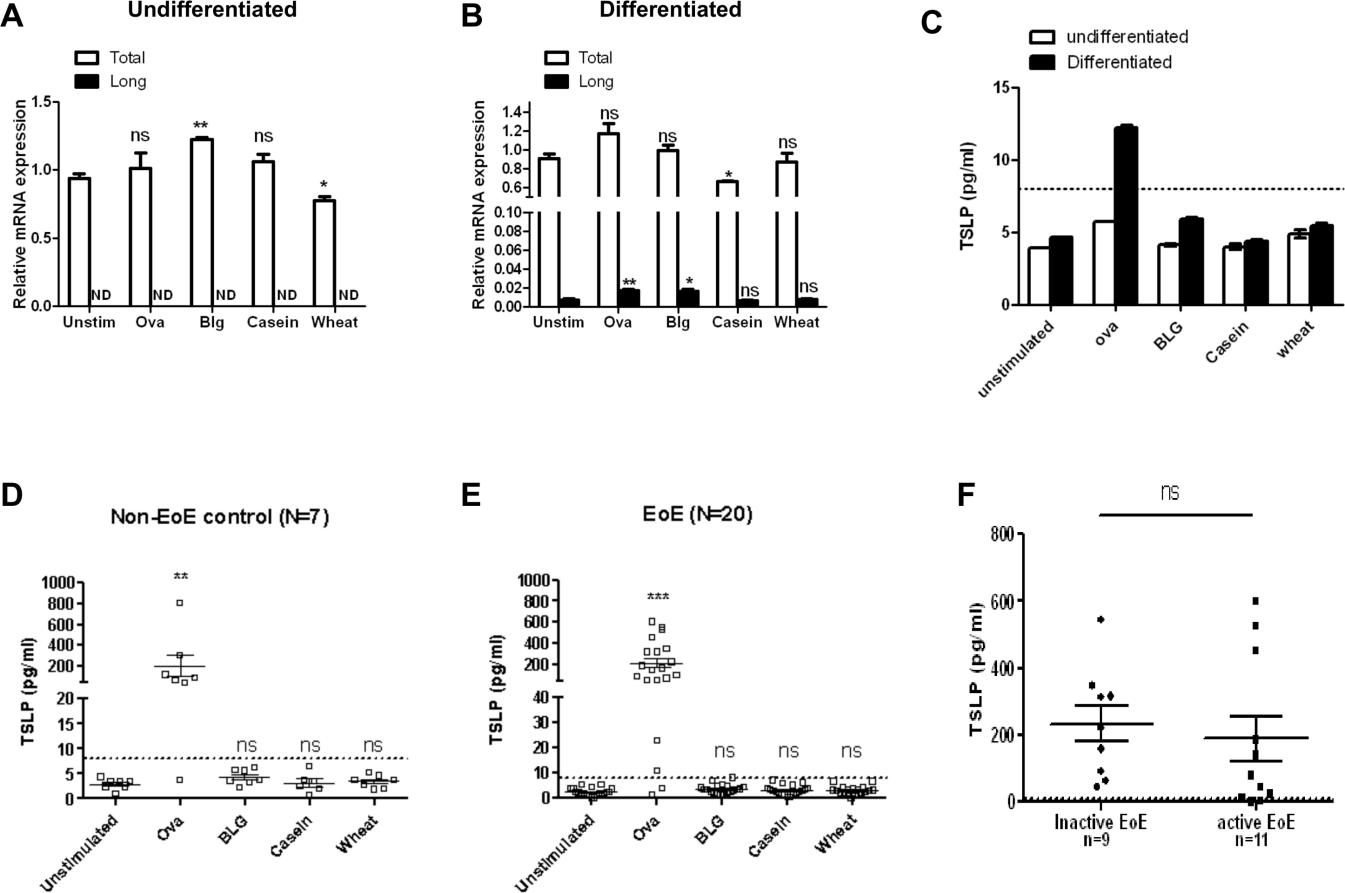
Food antigens induce esophageal epithelial TSLP expression. Quantification of TSLP mRNA expression following food antigen stimulation in undifferentiated **(A)** and differentiated **(B)** EPC2-hTERT cells. **C**) TSLP secretion (pg/mL) was quantified in undifferentiated and differentiated EPC2-hTERT cells. TSLP secretion in differentiated primary esophageal epithelial cells from non-EoE control subjects (N = 7) **(D)** and EoE subjects (N = 20) **(E)** following stimulation with food antigens. All cell lines were stimulated for 24 hours. (**F**) comparison of ova mediated TSLP expression in differentiated inactive and active EoE cells. The dashed lines represent the lower limit of sensitivity of the ELISA kit (8pg/mL). Bars represent the mean of each data set. * p<0.05, **p<0.01, ***p <0.001 as compared to unstimulated controls.

The six-food elimination diet (SFED) is effective in EoE and is based upon the prevalence of cow-milk protein, wheat, egg, peanut, seafood, and soy as causative antigens in EoE.[19]. Based upon preferential expression of TSLP within the differentiated compartment of the esophageal epithelium in EoE (**Fig 1**) and our findings that calcium differentiation enhances inducible TSLP expression, we hypothesized that TSLP might be induced by direct contact between food antigens and differentiated esophageal epithelial cells at the luminal surface of the esophagus. To investigate this *in vitro*, we stimulated EPC2-hTERT cells with individual food antigens, and compared the inducible mRNA expression and protein secretion of TSLP between differentiated and undifferentiated esophageal epithelial cells. Epithelial cells were stimulated with several food antigens from the SFED, including two cow milk proteins [β-lactoglobulin (BLG) and β-casein], chicken egg OVA, and wheat, the most commonly implicated food antigens in the SFED. While BLG induced a modest yet significant induction in the total TSLP transcript, none of the food antigens induced the long TSLP isoform in undifferentiated epithelial cells (**Fig 4A**). More importantly, food antigens failed to induce TSLP secretion in undifferentiated epithelial cells. (**Fig 4C white bars**). However, both OVA and BLG induced expression of the long TSLP transcript in differentiated EPC2-hTERT cells (**Fig 4B**). Surprisingly, OVA, but not BLG, induced TSLP protein secretion in differentiated EPC2-hTERT cells (**Fig 4C black bars**). We again verified these findings using and evaluated the difference between the short and long isoforms in differentiated cells stimulated with food antigens (**S3 Appendix**).

We next determined whether food antigens might induce epithelial TSLP secretion in primary esophageal epithelial cells (EPCs) using primary human EPCs cultivated from seven control (non-EoE) subjects and 20 EoE subjects. Similar to EPC2-hTERT cells, OVA stimulation of undifferentiated primary cell lines did not lead to detectable TSLP secretion (data not shown). In contrast, nearly all differentiated primary cell lines from EoE (18/20) non-EoE (6/7) cell lines secreted detectable TSLP in response to OVA stimulation (**Fig 4D** and **Fig 4E**). Differences between overall OVA-induced responses between EoE and non-EoE cohorts were not statistically significant. Comparisons of OVA-induced TSLP responses between cell lines obtained at the time of active EoE inflammation were similar to epithelial responses from cell lines from inactive EoE subjects (**Fig 4F**). Patient characteristics and *in vitro* responses are summarized in **Table 1**.

**Table 1:** Patient demographics, and allergy test results.

ID	Subject ID	Age	Gender	EGD Indication	Diagnosis	Status	Eos/hpf
**C01**	**131**	9 y 0 m	F	abdominal pain, diarrhea	control	Control	0
**C02**	**203**	15 y 3 m	F	abdominal pain	control	Control	0
**C03**	**425**	12 y 3 m	M	abdominal pain	control	Control	0
**C04**	**430**	15 y 6 m	F	GERD	control	Control	0
**C05**	**467**	5 y 4 m	M	dysphagia	control	Control	0
**C06**	**431**	1 y 6 m	F	vomiting	control w/FA	Control	0
**C07**	**515**	7 y 1 m	M	GERD, Food allergies	control w/FA	Control	0
**E01**	**151.5**	6 y 3 m	F	surveillance EGD, no PPI	EoE	Active	15
**E02**	**312**	15 y 5 m	F	avoiding soy, nuts, eggs	EoE	Active	15
**E03**	**434**	5 y 4 m	M	avoiding egg, milk, peanuts	EoE	Active	80
**E04**	**128**	4 y 6 m	M	added dairy	EoE	Active	75
**E05**	**141**	8 y 4 m	F	removed green beans	EoE	Active	20
**E06**	**397**	14 y 11 m	M	removed milk	EoE	Active	45
**E07**	**188.5**	10 y 5 m	M	removed milk	EoE	Active	50
**E08**	**280**	12 y 11 m	M	dysphagia	EoE	Active	50
**E09**	**394**	11 y 4 m	M	open diet	EoE	Active	37
**E10**	**443.3**	6 y 6 m	M	removed milk and added soy	EoE	Active	40
**E11**	**468**	16 y 0 m	M	open diet	EoE	Active	25
**E12**	**185.4**	7 y 2 m	M	added baked milk	EoE	Inactive	0
**E13**	**454.2**	11 y 6 m	M	avoiding milk	EoE	Inactive	3
**E14**	**116**	6 y 6 m	M	added squash	EoE	Inactive	0
**E15**	**196.6**	9 y 1 m	M	added beef on swallowed budesonide	EoE	Inactive	0
**E16**	**348**	11 y 9 m	F	restricted diet	EoE? Reflux and FA?	Inactive	1
**E17**	**368.2**	11 y 9 m	M	removed milk, wheat, chicken	EoE	Inactive	0
**E18**	**171.7**	10 y 8 m	M	removed grape jelly and cinnamon	EoE	Inactive	0
**E19**	**291**	16 y 8 m	M	open diet	EoE	Inactive	10
**E20**	**342**	5 y 10 m	M	added oat and potato	EoE	Inactive	0

IgE-mediated foods indicate antigens with positive skin prick test. **EoE foods by biopsy** include foods which, after addition or removal, led to exacerbation or improvement in esophageal eosinophilia after follow-up biopsy, respectively. **Disease status** reflects number of eosinophils per hpf at the time of biopsy; active (≥15 eos per hpf) or inactive (<15 eos per hpf).

### OVA-induced esophageal epithelial TSLP induction is dependent upon NF-kB signaling and inhibited by budesonide *in vitro*

**Fig 5:**
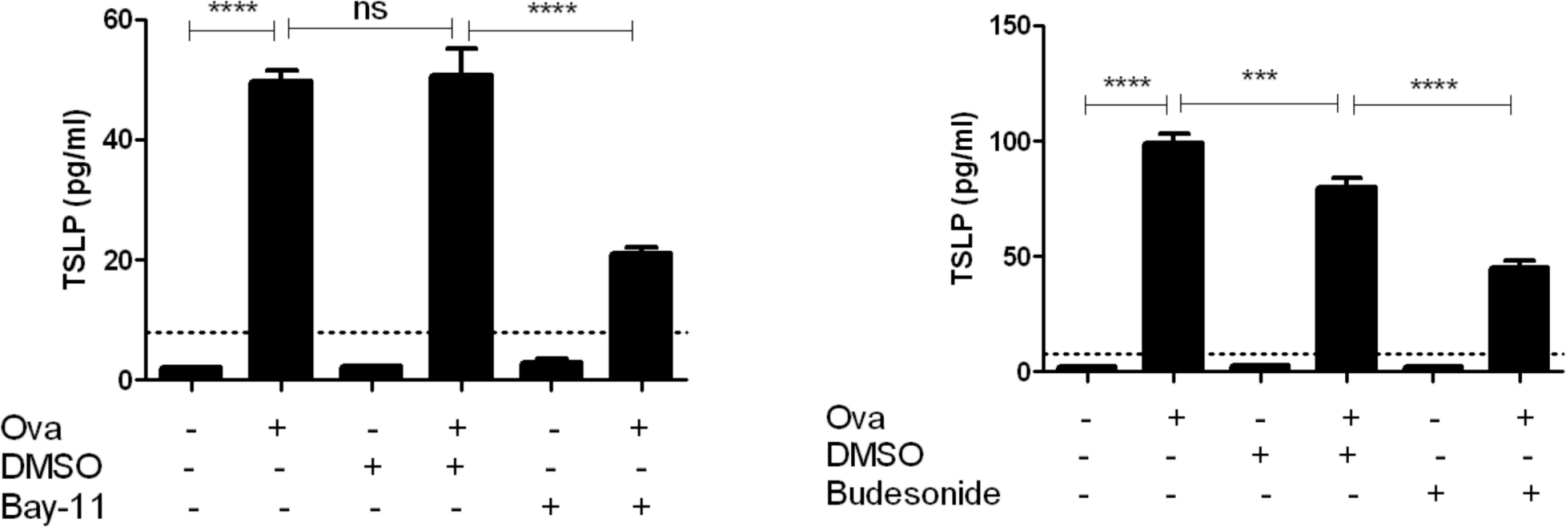
OVA-Induced esophageal epithelial TSLP induction is dependent upon NF-kB signaling and inhibited by budesonide *in vitro*. **A**) differentiated EPC2-hTERT cells were pre-incubated with Bay-11 for 1hr prior to stimulation with OVA. TSLP in culture supernatant was assayed after 24 hrs. **B**) Differentiated EPC2-hTERT cells were stimulated with Ova in the presence or absence of budesonide (1uM) or DMSO (vehicle) for 24hrs.TSLP in culture supernatant was assayed using ELISA. ***p<0.001, ****p<0.0001, NS = not significant.

TSLP expression is regulated in part by the transcription factor NF-kb ^38^. We determine the role of NF-kB signaling in OVA-mediated TSLP induction by stimulating differentiated EPC2-hTERT cells with OVA in the presence and absence of Bay-11 (10μM in DMSO), an irreversible inhibitor of NF-kB signaling. In the presence of Bay-11, OVA mediated induction of TSLP secretion was significantly suppressed (**Fig 5A**).

In addition to food elimination diets, swallowed steroids, including viscous solutions of budesonide, are effective treatments for EoE [27,29,42]. Swallowed “topical” corticosteroids with low systemic bioavailability may reduce inflammation through physical contact with the esophageal epithelium. To test this in our model system, we stimulated differentiated EPC2-hTERT cells with OVA in the presence and absence of 1μM budesonide(in DMSO). Co-treatment with budesonide robustly suppressed OVA induced TSLP secretion (**Fig 5B**).

## Discussion

We have shown that TSLP expression during active EoE is restricted to the suprabasal layer of the esophageal epithelium. We further show that differentiated esophageal epithelial cells, phenotypically similar to cells at the esophageal barrier surface, have altered innate immune responses compared to undifferentiated epithelial cells. Our study is the first to show differential responses to food antigen stimulation by human esophageal epithelial cells, and proposes a potential mechanism by which specific food antigens may incite host immune responses in EoE at the epithelial barrier.

TSLP expression is well documented at barrier surfaces where it may aid in host protection against pathogens and foreign antigens. This is evident from the ability of pathogens to induce TSLP expression by epithelial cells. Taylor et al showed constitutive TSLP expression in murine GI tract, and demonstrated that TSLP-TSLPR interactions were critical for host protection against intestinal nematode infection.[43] Additionally TSLP expression is induced at the barrier surface during allergic inflammation such as keratoconjuctivitis and atopic dermatitis [17,26]. Recently Fornasa et al described the short and long forms of TSLP in the context of inflammatory disease of the intestine and skin[4]. Interestingly, they found that the long form of TSLP was associated with inflammatory conditions and the short form was homeostatic. Our data support the findings in that the long form is induced in the setting of stimulation, with a robust stimulation of the long form in stimulated cells. Future work will focus on the role of the long form of TSLP in the esophagus *in vivo* and determine its functional role in response to allergic stimulation.

Laser capture microdissection (LCM) studies of the human epidermis have identified significant differences in gene expression patterns between basal and suprabasal epithelial cells. [44,45] Additionally, *in vitro* differentiation of airway epithelial cells leads to enhanced TLR3 expression and resistance to rhinovirus infection. [46,47] Similar to airway epithelial cells, terminal differentiation of esophageal epithelial cells resulted in enhanced TLR3 expression and TLR3-mediated TSLP secretion and demonstrates that differentiated esophageal epithelial cells have altered innate immune responses compared to undifferentiated cells.

Though clinical practice demonstrates that food antigens drive EoE disease pathogenesis, the mechanisms by which this occurs are unclear. In murine EoE models, esophageal eosinophilia occurs after systemic allergen sensitization, supporting the notion that EoE is a localized response to systemic food allergen sensitivity. In clinical practice, reductions in esophageal eosinophilia can be achieved by administration of elemental formulas via nasogastric or gastrostomy tubes,[21] bypassing direct contact with the esophageal epithelium. In contrast, the clinical efficacy of swallowed corticosteroids with low bioavailability[42] supports an inciting role for esophageal epithelial cells in EoE host responses. As additional support for the role of epithelial cells in EoE immune responses, Masterson et al. described a murine EoE model (L2-IL5) in which esophageal epithelial cells overexpress IL-5 [48]. Compared to other models in which eosinophils are primarily recruited to the lamina propria, the L2-IL5 model is the first murine model with significant intraepithelial eosinophilia, underscoring the importance of esophageal epithelial-derived cytokine expression in EoE pathogenesis. While our data suggest that esophageal epithelial cells may be involved in host inflammatory responses to specific environmental stiumuli, it is likely that both the systemic and local immune responses contribute to the complex host response to food antigens in EoE.

OVA is a classic antigen used to study immune tolerance, antigen presentation, and allergic responses *in vitro* and *in vivo*. Both acute and chronic sensitization with OVA is known to recapitulate key features of clinical asthma in murine models including eosinophilia and goblet cell hyperplasia [49] We now show that differentiated esophageal epithelial cells secrete TSLP in response to OVA challenge *in vitro* via unknown mechanisms. We speculate that undifferentiated epithelial cells in the basal compartment may act as non-professional antigen presenting cells as observed by Mulder et al, while the terminally differentiated cells at the barrier layer are involved in TSLP secretion thus essentially bridging the innate-adaptive immune responses. Additionally, contribution of the pro-inflammatory environment on epithelial antigen response cannot be ignored as they have been shown to enhance peptide internalization in other models[50]

Though cow milk proteins are the most common dietary trigger for pediatric EoE, [22], neither βLG nor casein significantly induced epithelial expression of TSLP. It is possible that cow milk proteins induce the epithelial expression of other EoE-associated cytokines, as TSLP is one of many EoE-associated cytokines. Alternatively, while OVA may induce TSLP through direct epithelial contact, other food antigens, including cow milk proteins, may require intestinal absorption and subsequent processing by antigen presenting cells (APCs) with the gut associated lymphoid tissue (GALT), as a prerequisite to host systemic Th2 priming. An additional possibility is that other food antigens including cow milk protein may gain access to resident esophageal epithelial dendritic cells and T cells via enhanced epithelial paracellular permeability, which has been proposed in models of food allergy [51,52].

In this study, we found that the differentiation characteristics and innate immune responses of primary esophageal epithelial cells from EoE subjects was not distinct from that of non-EoE cell lines *in vitro*. Furthermore, there was no correlation between individual patient allergies and their TSLP response to those specific foods. These findings are in keeping with previous studies in which epithelial cells from EoE, and non-EoE subjects were found to exhibit similar growth and functional characteristics *in vitro* [33,53]. We show that esophageal epithelial cells from EoE and non-EoE subjects have similar responses to OVA challenge *in vitro*, supporting the concept that downstream systemic immune responses are critical determinants of the host phenotype. It is plausible that innate esophageal epithelial responses to food antigens are down-regulated in the non-EoE host, whereas these responses may be may be amplified within the EoE host. Indeed, oral tolerance is an active immunologic process, rather than a lack of immune response to oral antigens [54–56]. Our findings, coupled with clinical observations that tolerance to EoE-trigger foods can develop in approximately 5% of EoE patients,^52^ highlights the complexity of the host immune response in EoE, and suggests that both epithelial immune responses and the host systemic response are involved in EoE pathogenesis. However, these *in vitro* studies, including the current study, are limited by a small sample size. Future larger studies, will be required to determine whether these results may be generalizable findings. Our small sample size also precludes a determination of whether in vitro food antigen stimulation correlates with clinical food antigen sensitivity in EoE, but may be a provocative avenue for future investigation.

Emerging evidence suggests that epithelial TLRs may be involved in environmental allergen mediated inflammation. Li et al showed that rag weed pollen mediated TSLP induction in corneal epithelial cells *in vitro* and *in vivo* through TLR4 signaling[57]. As TLR4 is neither expressed nor functional in either EPC2-hTERT cells or the parental EPC cell line [58], TLR4 is unlikely to play a role in inducible esophageal epithelial TSLP expression *in vitro*. This is consistent with the findings of Lee et al. who reported that induction of TSLP by bronchial epithelial cells is not induced by LPS [59]. While the mechanism by which OVA induces epithelial TSLP expression is unknown, a possible role for other TLRs in OVA-mediated epithelial TSLP expression should be further investigated. Together, these results underscore the complexity of epithelial innate immune response to environmental allergens and encourage future studies to understand the mechanism underlying food antigen mediated epithelial inflammatory responses in EoE.

Our results are in keeping with previous reports that NF-κB signaling regulates poly (I:C) and cytokine-mediated TSLP expression in various epithelial cell types[59,60]. Swallowed corticosteroids (budesonide and fluticasone) are commonly used as monotherapy or adjunctive therapy to dietary restriction in EoE and improves clinical and histological outcomes in both pediatric and adult populations[61,62]. *In vitro*, budesonide suppresses allergen induced cytokine secretion from epithelial cells and fibroblasts [63,64]. We now show that budesonide can suppress inducible TSLP secretion in esophageal epithelial cells *in vitro*, thus offering a plausible new mechanism for budesonide mediated anti-inflammatory effect.

In summary, our study shows that TSLP is predominantly expressed in the differentiated compartment of the esophageal epithelium in EoE and this can be recapitulated *in vitro* in calcium based terminal differentiation model. More importantly, our observations of OVA-mediated TSLP expression from differentiated epithelial cells offer novel evidence in support of the crucial role of esophageal epithelium in the pathogenesis of EoE.

## Supporting Information

S1 Appendix TSLP expression increases with increasing concentrations of OVA. Differentiated EPC2-hTERT cells were stimulated with increasing concentrations of OVA. *, p<0.05.
(TIFF)

S2 Appendix Expression of short and long isoform of TSLP in differentiated EPC2-hTERT cells stimulated with poly(IC). *,p<0.05.
(TIFF)

S3 Appendix Expression of short and long isoform of TSLP in differentiated EPC2-hTERT cells stimulated with food antigens for 3 hours. *, p<0.001.
(TIFF)
