# Supplementary figures and images for: Preferential Secretion of Thymic Stromal Lymphopoietin (TSLP) by Terminally Differentiated Esophageal Epithelial Cells: Relevance to Eosinophilic Esophagitis (EoE)
Source: PLoS One. 2016 Mar 18;11(3):e0150968. doi: 10.1371/journal.pone.0150968 (PMC4798725; doi:10.1371/journal.pone.0150968)

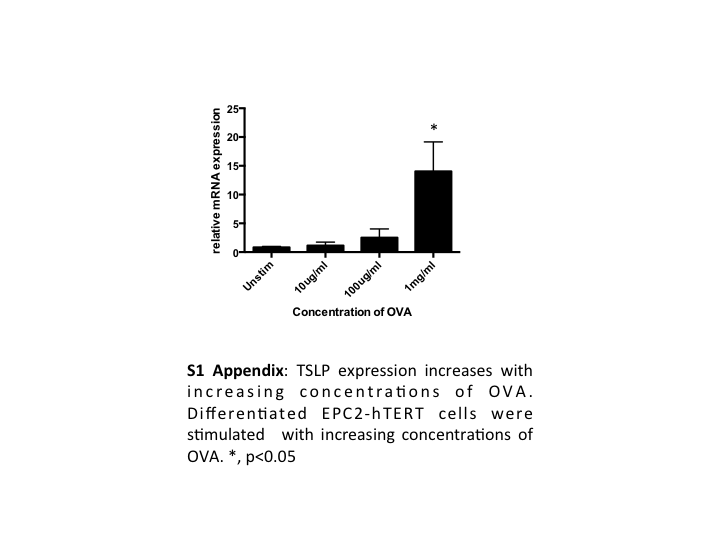

Supplement: S1 Appendix — Differentiated EPC2-hTERT cells were stimulated with increasing concentrations of OVA. *, p<0.05. (TIFF) [file pone.0150968.s001.tiff]

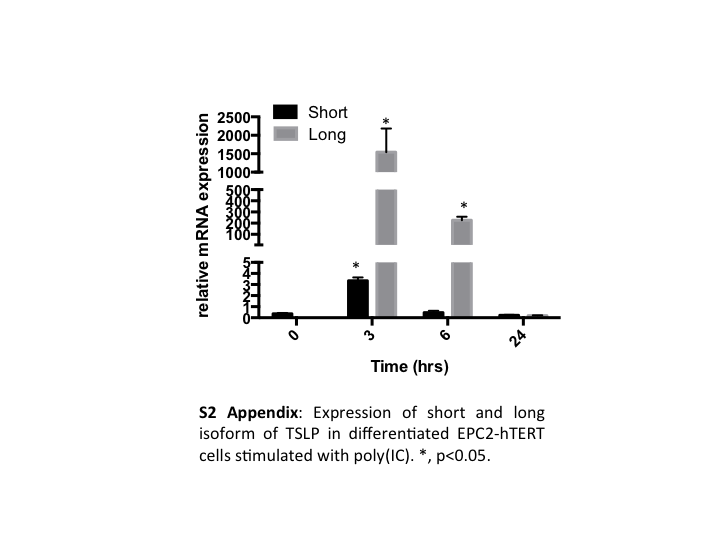

Supplement: S2 Appendix — *,p<0.05. (TIFF) [file pone.0150968.s002.tiff]

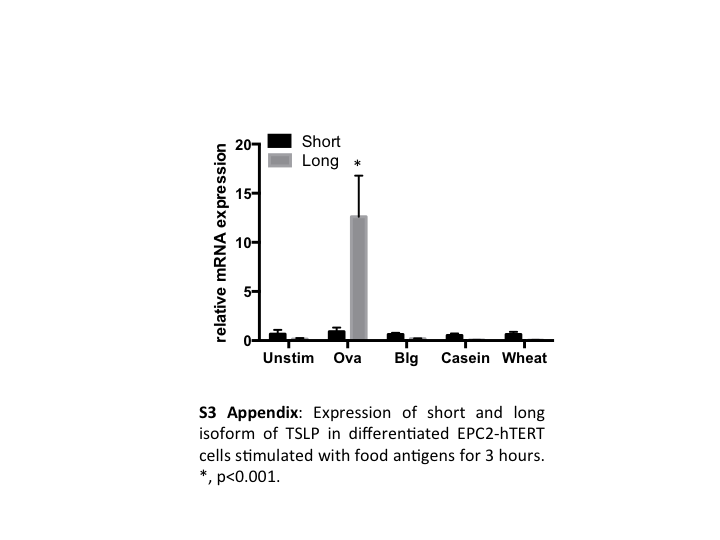

Supplement: S3 Appendix — *, p<0.001. (TIFF) [file pone.0150968.s003.tiff]
